# Semi-automated title-abstract screening using natural language processing and machine learning

**DOI:** 10.1186/s13643-024-02688-w

**Published:** 2024-11-01

**Authors:** Maximilian Pilz, Samuel Zimmermann, Juliane Friedrichs, Enrica Wördehoff, Ulrich Ronellenfitsch, Meinhard Kieser, Johannes A. Vey

**Affiliations:** 1 University of Heidelberg - Institute of Medical Biometry, Heidelberg, Germany; 2 Fraunhofer Institute for Industrial Mathematics - Department of Optimization, Kaiserslautern, Germany; 3 Medical Faculty of the Martin Luther University Halle-Wittenberg - Department of Visceral, Vascular and Endocrine Surgery, Halle (Saale), Germany

**Keywords:** Machine learning, Natural language processing, Language models, Systematic review, Meta analysis, Automatization, Title-abstract screening

## Abstract

**Background:**

Title-abstract screening in the preparation of a systematic review is a time-consuming task. Modern techniques of natural language processing and machine learning might allow partly automatization of title-abstract screening. In particular, clear guidance on how to proceed with these techniques in practice is of high relevance.

**Methods:**

This paper presents an entire pipeline how to use natural language processing techniques to make the titles and abstracts usable for machine learning and how to apply machine learning algorithms to adequately predict whether or not a publication should be forwarded to full text screening. Guidance for the practical use of the methodology is given.

**Results:**

The appealing performance of the approach is demonstrated by means of two real-world systematic reviews with meta analysis.

**Conclusions:**

Natural language processing and machine learning can help to semi-automatize title-abstract screening. Different project-specific considerations have to be made for applying them in practice.

**Supplementary Information:**

The online version contains supplementary material available at 10.1186/s13643-024-02688-w.

## Background

Collecting knowledge from different studies by combining them within a systematic review with or without meta analysis is an important contribution to the generation of high-level evidence in medicine. This evidence is often used for the development of trustworthy guidelines and to inform policy makers, health care providers, and patients [1]. However, collecting the information included in different studies is a time-consuming task and the amount of biomedical literature is growing. The comprehensive literature search should be as extensive as possible to identify all relevant studies and to reduce the risk of reporting bias. As a consequence, thousands of citations matching the search criteria may be found. Subsequently, these citations need to be screened in two stages with regard to the inclusion and exclusion criteria to answer a particular medical research question. In a first stage, the abstracts of all identified studies are screened and determined to be relevant or not. In the second stage, the full texts of the relevant studies are assessed regarding inclusion in the systematic review meeting the specific criteria.

The average time from registration to publication of a systematic review is 67 weeks [2] even though no patient data has to be collected but only existing studies are screened and summarized. Due to the importance of systematic reviews for evidence-based medicine and the large amount of work required, there are approaches to (semi-)automate various processes including the search of randomized controlled trials (RCT), screening of citations, data extraction, and bias assessment [3]. In particular, title-abstract (TIAB) screening is a time-consuming task since it is common practice that at least two human reviewers independently read all identified titles and abstracts and decide whether the respective citation has to be included or excluded from full-text screening. Disagreement is commonly solved by a third person. However, this task consists of plain text reading and could thus potentially be done by methods of natural language processing (NLP) and machine learning (ML). More specifically, a combined text processing and ML pipeline could be established to, first, convert titles and abstracts into a dataset that is suitable for ML and, second, build a classifier to the classification problem “include/exclude.”

Some existing software tools support the TIAB screening process for systematic reviews in medical research [4]. Those are, for example, Abstractr [5], RobotReviewer [6], EPPI Reviewer [7], RobotAnalyst [8], SWIFT-Review [9], Colandr [10], Rayyan [11], DistillerSR [12], and ASReview [13]. Typically, these tools process the titles and abstracts using NLP and subsequently train an ML algorithm to classify additional publications. These tools share a common feature: they train multiple ML models adaptively. This is achieved by continually augmenting the training dataset with publications identified as most relevant by the ML algorithm, a process known as active learning [13]. Consequently, the tool engages with reviewers during the screening process, iteratively recommending the next papers to be reviewed. However, this approach may be inconvenient in practice, as screeners may find it uncomfortable to frequently pause their workflow to await the ML model’s recommendation for the next paper to screen. A great advantage of these tools is their easy usage for researchers without strong programming skills and their high performance in applications. They could reduce the workload with minimal risk of excluding relevant citations. However, these approaches are typically inflexible and cannot be adapted to specific demands. Evaluation studies showed that their performance greatly varied when applied to different types of reviews [14–16]. Furthermore, only one classification algorithm is trained based on a specific data representation. It has been shown that the performance of a classifier depends on the processing of the titles and abstracts and the choice of the model [17]. Lange et al. [17] compared various methods by means of diagnostic test studies, and other approaches focused, e.g., on pre-clinical animal studies [18], *in vitro* studies [19], or cluster randomized trials [20]. Recently, Kebede et al. [21] evaluated the performance of NLP and ML algorithms for TIAB screening.

These tools can be subsumed into two categories. First, there are proof-of-concept publications which show that ML can help with TIAB screening but do not provide guidance on how to apply the procedures in practice. Second, complete software packages are made available that allow the application of NLP and ML techniques to TIAB screening. While these packages can be enormously helpful, they are hardly adjustable. This means that the source code can be modified only with much effort (or not at all) and this complicates their applicability in situations that deviate from the intended use case. Examples of such deviations may be another data coding or the desire to apply other than the implemented NLP or ML techniques. A detailed description of the necessary steps to apply NLP and ML techniques in practice together with easy-to-apply source code and recommendations for practical use is still missing to the best of our knowledge.

In this paper, we present a semi-automated approach to TIAB screening, which is straightforward, transparent, and allows for customization in order to be adaptable to different scopes of systematic reviews. This makes the presented methodology of particular interest for statisticians and other programmers who work on the task to automatize steps of TIAB screening by ML techniques. We describe in detail the text processing that converts the titles and abstracts into a dataset which is subsequently used to train a selection of ML algorithms. The resulting dataset is split into a training and a test set. For the training set, the include/exclude decision is made by two human screeners. Different ML models are trained on this training data and evaluated on the test set. We iteratively grow the training set in order to investigate the required minimum training set size after which at least one of the human screeners can stop their involvement. This procedure is described in the “Methods” section. We present the application of our approach to the systematic reviews by Friedrichs et al. [22] and Wadewitz et al. [23] in the “Results” section. In the “Practical implications” section, we elaborate in more detail how the approach can be applied to a concrete practical setting. In the “Discussion” section, we conclude with a discussion, and give an outlook for future application and research on the presented approach.

## Methods

**Fig. 1:**
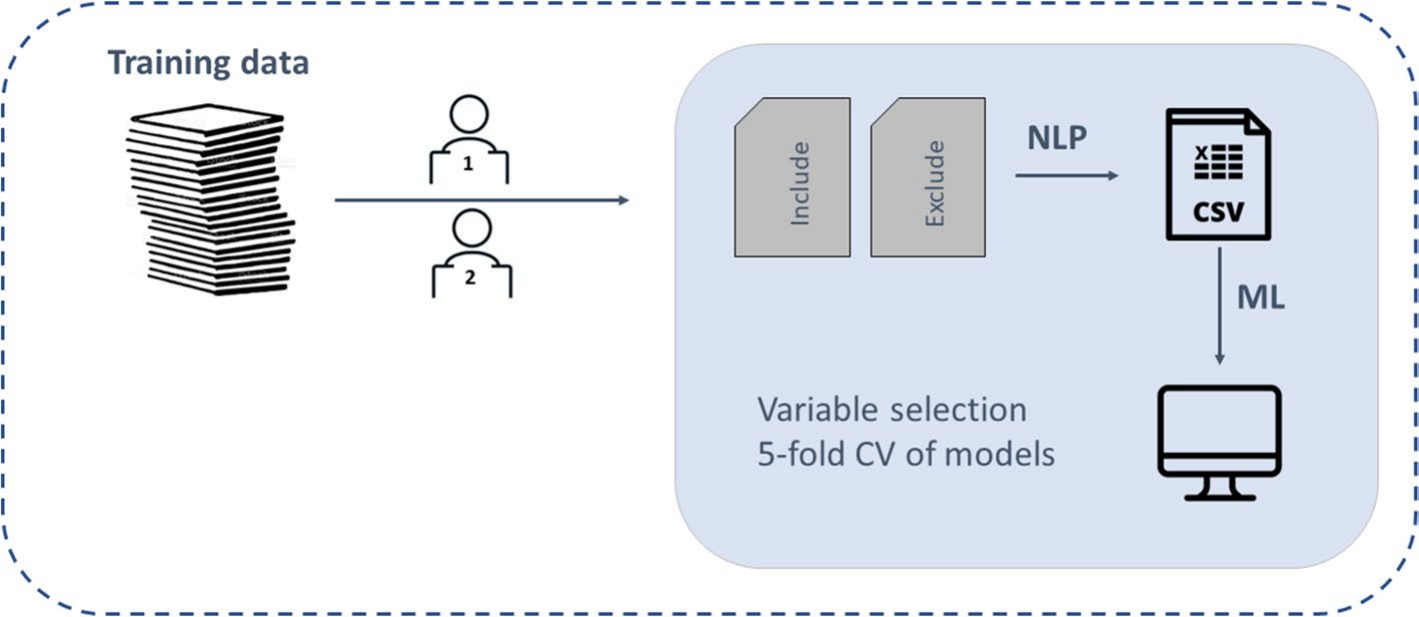
Workflow of presented methodology. First, the title and abstracts are successively screened by two human reviewers who decide if a citation should be included or excluded. Next, the titles and abstracts are processed by natural language processing (NLP) techniques to receive a numeric dataset, that is subsequently used to train machine learning (ML) models with respect to binary classification of in- or exclusion

The methodology of the presented classification approach is summarized in Fig. 1. In the following subsections, the respective steps are described in detail.

### Datasets

Two different datasets from real systematic review projects were used regarding the development and evaluation of the approach of semi-automated TIAB screening.

#### INTRISSI

The presented approach was first developed and evaluated within the systematic review with meta analysis by Friedrichs et al. [22] from the INTRISSI project. The objective of this systematic review is to assess the relative contribution of intravenous antibiotic prophylaxis, mechanical bowel preparation, oral antibiotic prophylaxis, and combinations thereof towards the reduction of surgical site infection incidence in elective colorectal resections. Literature search was performed in several databases, including Cochrane Central Register of Controlled Trials (CENTRAL), Cochrane Database of Systematic Reviews (CDSR) from The Cochrane Library, MEDLINE, LILACS (Literatura Latinoamericana y del Caribe en Ciencias de la Salud), Current Contents/Clinical Medicine, and Web of Science. The systematic review was limited to randomized controlled trials (RCT). The identified citations were imported to the software Rayyan [11] for the manual screening. Duplicates were removed and the citations were exported as CSV files to be used for the automated TIAB screening.

#### PPU

The approach was applied and evaluated in a further real systematic review about the comparison of surgical and alternative approaches for the treatment of perforated peptic ulcers (PPU) [23]. The literature search was conducted in PubMed, Cochrane Library, Embase, Cumulative Index to Nursing and Allied Health Literature (CINAHL), Clinical.Trials.gov, and International Clinical Trials Registry Platform (ICTRP). Again, the identified citations were imported to Rayyan [11], screened according to in- and exclusion criteria, and exported as CSV files.

### Text processing

The unstructured titles and abstracts had to be transformed into a data frame that allows the application of ML methods. We aimed at creating a data frame with one row per publication and one column per word (unigram). The cells should show the counts how often the respective word appeared in the corresponding title and abstract. To achieve this, NLP techniques were applied. First, all words were set to lower case. Then, the words were lemmatized. This means that words were reduced to their *lemmas*, thus their intended meaning. For instance, the words “analyze” and its progressive form “analyzing” were both lemmatized to “analyze” since they have the same meaning. Afterwards, to avoid special characters, all of them (as well as numbers) were deleted as well as any white space. To avoid that words with the same interpretation were treated as separate words, word stems were built using Porter’s stemming algorithm [24]. In a next step, we deleted fill words as, for instance, “and” or “also” as well as words that we did not expect to be useful for classification as, e.g., “england” or “https.” These words were detected by comparing the word stems to those of the list of Grady Ward’s English words [25].

At last, one comment to lemmatization and stemming is warranted. In NLP, often only lemmatization is used since it returns words that indeed exist (e.g., “history” instead of the stem “histori”). However, in our case, we focused on whether or not a word stem appeared in the title/abstract and, therefore, we only needed the word stems. Note that in those TIAB screening problems, there usually is a $p > n$ situation, i.e., there are many more words than included studies. We made the best experience with using lemmatization and stemming together since this reduces the number of different words and therefore the number of different columns in the dataset, mitigating the $p > n$ issue. To illustrate this effect, one can look at the words “program,” “programming,” and “programmer.” While lemmatization reduces the progressive form “programming” to “program,” the noun “programmer” remains unchanged. Stemming, however, converts all three words to “program.” In our case, we were not interested in differentiating whether someone is “programming” or whether is a “programmer.” The only fact of relevance was to know whether anything related to “program” appears in the text.

### Machine learning

#### Variable selection

Due to the fact that the number of columns was much larger than the number of rows ($p > n$), variable selection was applied. First, we decided to remove all words (i.e., columns) that appeared only once since they would not contribute to the final decision due to a missing second appearance. Second, we applied an elastic net [26] that is an $l_1$- and $l_2$-penalized logistic regression. The words that were selected by this procedure were used as candidate features for classification. Note that when the dataset was split into training and test set, the described variable selection procedure was only applied on the training set to avoid bias.

#### Machine learning methods

According to the no-free-lunch theorem [27], there is no learning method that generally performs well for every prediction problem. This implies that a new model comparison is necessary for each prediction task. To depict the variety of methods appropriately, we chose a kernel-based method (support vector machine), a linear-model-based approach (logistic regression), and two tree-based methods, among which one uses bagging (random forests) and one uses boosting (LightGBM) to combine multiple trees. In this section, the four candidate methods are briefly described and references are given. For a detailed description of statistical learning, we refer to the book by Hastie et al [28].

1. Support vector machines (SVMs) are a kernel-based learning method. The features are mapped by a kernel function into high-dimensional feature spaces to allow for non-linear classification boundaries in the decision space. The empirical representer theorem reduces the dimension of the optimization problem and with advanced optimization techniques, the optimal SVM can be computed for different loss functions. A mathematical description of SVMs is given by Steinwart and Christmann [29].
2. Logistic regression is a popular variant of generalized linear models. In comparison to linear models that are suitable for continuous data, logistic regression is used for classification. The linear predictor is mapped by a suitably chosen link function into the interval [0, 1], thus defining a probability. A general overview of generalized linear models is provided by McCullagh and Nelder [30].
3. Random Forest [31] is an ensemble method of decision trees that is based on bagging. This means that the trees are trained on bootstrapped versions of the dataset in order to reduce variance in the data. These trees are then united by majority vote. Thus, the class that is predicted by the majority of the trees is chosen as random forest prediction.
4. LightGBM [32] is an approach for gradient boosting of decision trees. By gradient boosting, its performance is improved in each iteration. Compared with the probably more famous XGBoost algorithm, it shows a remarkable reduction in runtime.

Apart from logistic regression, all these methods have different hyperparameters that have to be set. We defined the hyperparameters by grid search and five-fold cross-validation on the training set, thus the prediction of the validation set was entirely blinded. The concrete grids can be found in the code provided as supplemental material.

#### Performance measures

Since usually in TIAB screening the major part of publications is not forwarded to full-text screening, one has to deal with imbalanced data. In order to obtain a classification that is trained without focusing on a specific cutoff value for inclusion, the main performance measure is the area under the curve (AUC) under the receiver operating characteristic (ROC) curve. Confidence intervals for the AUC are computed by bootstrapping. Since it is more important to include all publications that should be included to full-text screening than excluding all non-relevant papers, a cutoff value will be defined. Publications with a larger to-be-included probability than this cutoff will be included in the full-text screening and publications with a lower probability excluded. The proportion of correctly included papers among all included papers is denoted as true-positive rate. Analogously, the proportion of correctly excluded papers among all excluded papers is called true-negative rate.

To investigate the number of training samples that are needed to provide an acceptable prediction quality we applied the following procedure. The dataset was split into training and test sample. The size of the training sample was iteratively increased from 10% of the full dataset to 90% of the full dataset in steps of 10%. For each resulting classification problem, the variable selection procedure was performed and the ML methods were trained on the training set. They were evaluated on the test set by computing the AUC.

#### Cutoff value

Furthermore, a cutoff value was determined that could be used to decide whether or not a publication should be forwarded to full-text screening. Since the dataset is expected to be imbalanced (more publications are excluded from than included into full-text screening), special techniques are necessary. In general, one may optimize the Youden index or the geometric mean of sensitivity and specificity to obtain a “best” cutoff value. However, in the context of TIAB screening, mistakenly excluding relevant publications is worse than including too many publications since the first error cannot be corrected during full-text screening. Therefore, we propose the following approach to compute the cutoff value. We applied cross-validation on the training set since in practice the results of the test set are not known. In each iteration of the cross-validation, the training set was split into two subsets. On the first subset, the model was trained and the probabilities for inclusion of publications in the second subset were predicted by this model. The cutoff value was then defined as the smallest to-be-included probability among all publications in the second subset that were included to full-text screening by human screeners. For *K*-fold cross-validation, this yields *K* cutoff values. As final cutoff, one could for instance use the minimum, the median, or the mean of the *K* cutoff values, depending on how secure one wants to be to not miss important publications for full-text screening. These approaches will be explored in the “Choice of cutoff value” section.

### Fine-tuned language models

Currently, language models are gaining more and more attention. Those are neural-network-based text models which are trained on huge amounts of data to learn patterns and generate coherent responses. To do text classification using language models, pretrained language models are fine-tuned by the training data to allow for classification. The titles and abstracts are transferred to the models as a whole together with their respective label (include or exclude). Then the underlying model is updated in order to learn to distinguish between to-be-included and to-be-excluded publications.

An increasing number of language models exist but not all of them are freely available for fine-tuning. We applied two language models to our data. GPT-2 [33] is the most recent GPT-model that is freely available. Furthermore, we applied SciBERT [34], a variant of BERT [35] that is specifically trained for scientific text. Both are transformer-based models that differ in their architecture. While GPT-2 aims at predicting the next word in a sequence, given the previous words, BERT is designed to predict a missing word in a sentence by considering context from both left and right. The latter makes BERT, and consequently also SciBert, well-suited for the task of text classification while GPT-2’s main purpose is the generation of human-like text. However, it can be used for text classification as well. As the fine-tuning of language models takes a long time and therefore also consumes a lot of energy, cutoff values as described above were not computed for the language models. For the same reason, we did not perform any hyperparameter tuning for the language models. This of course gives a disadvantage to the language models compared with the ML algorithms. It should, however, be noted that hyperparameter tuning would costs many hours of runtime, making the approach unfavorable for application in practice.

### Software

Text processing was done using R, version 4.3.2 [36]. In particular, the packages textstem [37] and tm [38] were used for NLP techniques. The package qdapDictionaries [39] was used as a reference dictionary containing the list of Grady Ward’s English words. For data wrangling and plotting, the tidyverse [40] packages were applied. Machine learning models were trained using Python, version 3.11, in particular the scikit-learn package [41]. Language models were fine-tuned in Python, using the transfomers library [42]. R and Python were connected by means of the package reticulate [43]. A reproducible report based on both languages was built with Quarto (https://quarto.org/). Example code is available as supplemental material.

## Results

### INTRISSI

We used the research question by Friedrichs et al. [22] as an example, which was briefly outlined in the “INTRISSI” section. In the literature research, after removing duplicates, 4958 citations were detected among which 4460 were declared suitable for TIAB screening. Citations for which abstracts were not available or in another language than English were removed. The human screeners included 225 of these publications for full-text screening. After full-text screening, 128 citations remained for the systematic review. For the classification, 10164 word stems appeared at least twice in the titles and abstracts.

**Fig. 2:**
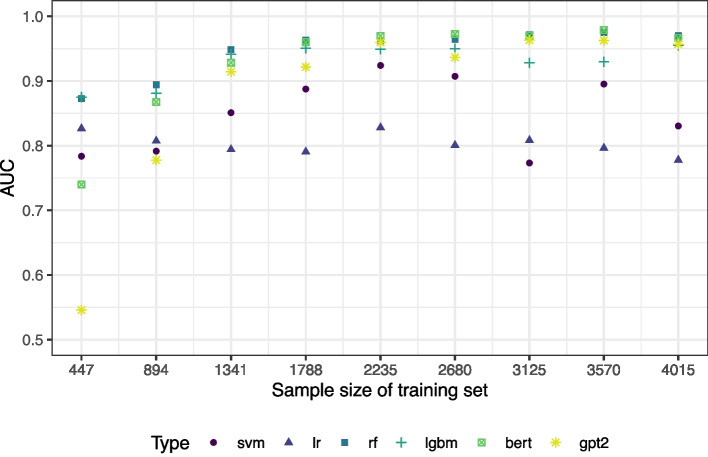
AUC values depending on the size of the training set

Next, the results are reported for iteratively increasing the training sample from 10% to 90% of the total dataset in steps of 10%. The included publications were distributed uniformly to the 10% batches, this means that also the number of to-be-included publications continuously increases with an increasing training sample. The AUC values for each method are visualized in Fig. 2. The corresponding 95% confidence intervals are provided in Table 1 in the Appendix.

We observe that the larger the training sample, the better the performance of the complex methods random forest, LightGBM, and support vector machine. This shows once more that machine learning methods need a certain amount of data to be able to outperform classical methods such as logistic regression. The AUC values were above 87% for the random forest even with a training sample of only 447 publications. The results remained quite stable from a training proportion of 1341 publications on. The only exception was the SVM at a training size of 3125 publications where the performance dropped remarkably. This illustrates that in the current dataset, a training set of 1341 publications would have been sufficiently large to predict the remaining papers’ allocation. The language models SciBERT and GPT-2 perform weak for a small amount of training data. From a training size of 1341 on, they perform equally well as the tree-based methods random forest and LightGBM. However, they do not outperform these algorithms. It should be recalled that no hyperparameter tuning was performed for the language models for runtime reasons.

### PPU

To explore our findings on a second dataset, we investigated the systematic review PPU as outlined in the “PPU” section. In the literature research, 1343 publications were declared suitable for TIAB screening. The human screeners included 79 of these publications for full-text screening. For the classification, 4370 word stems appeared at least twice in the titles and abstracts.

**Fig. 3:**
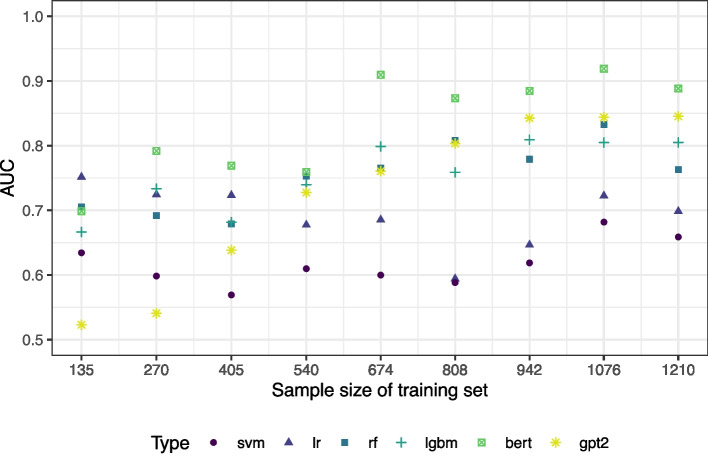
AUC values depending on the size of the training set

Figure 3 shows the results depending on the size of the training data. In the Appendix, Table 2 gives the corresponding 95% confidence intervals. In this example, larger sample sizes are needed to achieve an AUC of more than 80%. Furthermore, the finding that logistic regression and SVMs do not perform on an equivalent level as the other algorithms is confirmed here. From a training sample of around 1000 publications on, the language models (again without hyperparameter tuning) are the best-performing methods. Particulary the SciBERT algorithm outperforms all other methods on this dataset. AUC values in the range of more than 90% as in the INTRISSI example are only observed for SciBERT. Note, however, that the total sample size was smaller here.

### Choice of cutoff value

**Fig. 4:**
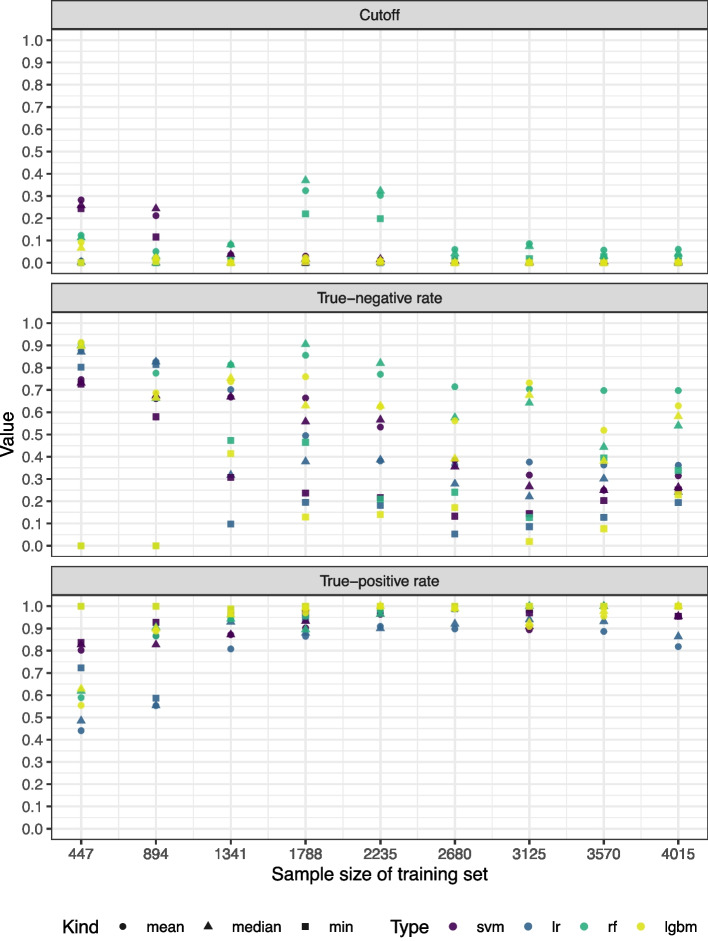
Cutoff, true-negative rate, and true-positive rate depending on size of the size of the training set

For the systematic review INTRISSI, we analyzed the cutoff values that are defined only on the training set in order to decide which publications from the test set should be included into full-text screening. Besides the cutoff, also the corresponding true-positive and true-negative rates are reported. These three numbers are depicted in Fig. 4 for each learning method and when choosing the final cutoff as mean, median, or minimum, respectively, of the cutoff values that were obtained by cross-validation. As mentioned in the “Fine-tuned language models” section, the cutoff values were not computed for the language models due to an enormous amount of runtime and resources that would have been necessary.

We observed that the true-positive rates were in a high range from training samples of 1341 publications onwards when the learning method was a random forest or LightGBM. Especially the random forest also showed high true-negative rates. This observation is in line with the AUC values that were highest for the tree-based approaches random forest and LightGBM. There were no large differences between choosing the mean or the median of the cutoff values obtained from cross-validation. Choosing the minimum increased the true-positive rate by decreasing the true negative rate as expected. Thus the minimum reduced the probability of missing important publications for full-text screening by increasing the number of to-be-excluded publications that were falsely included into full-text screening. The true-negative rates were not monotonic in the proportion of the training set. This could hold true since we randomly chose the publications to be added to the training set iteratively and thus it may have happened that in one iteration more or less complicated cases switch from test to training set, making the classification more or less difficult, respectively.

## Practical implications

### Extended workflow

**Fig. 5:**
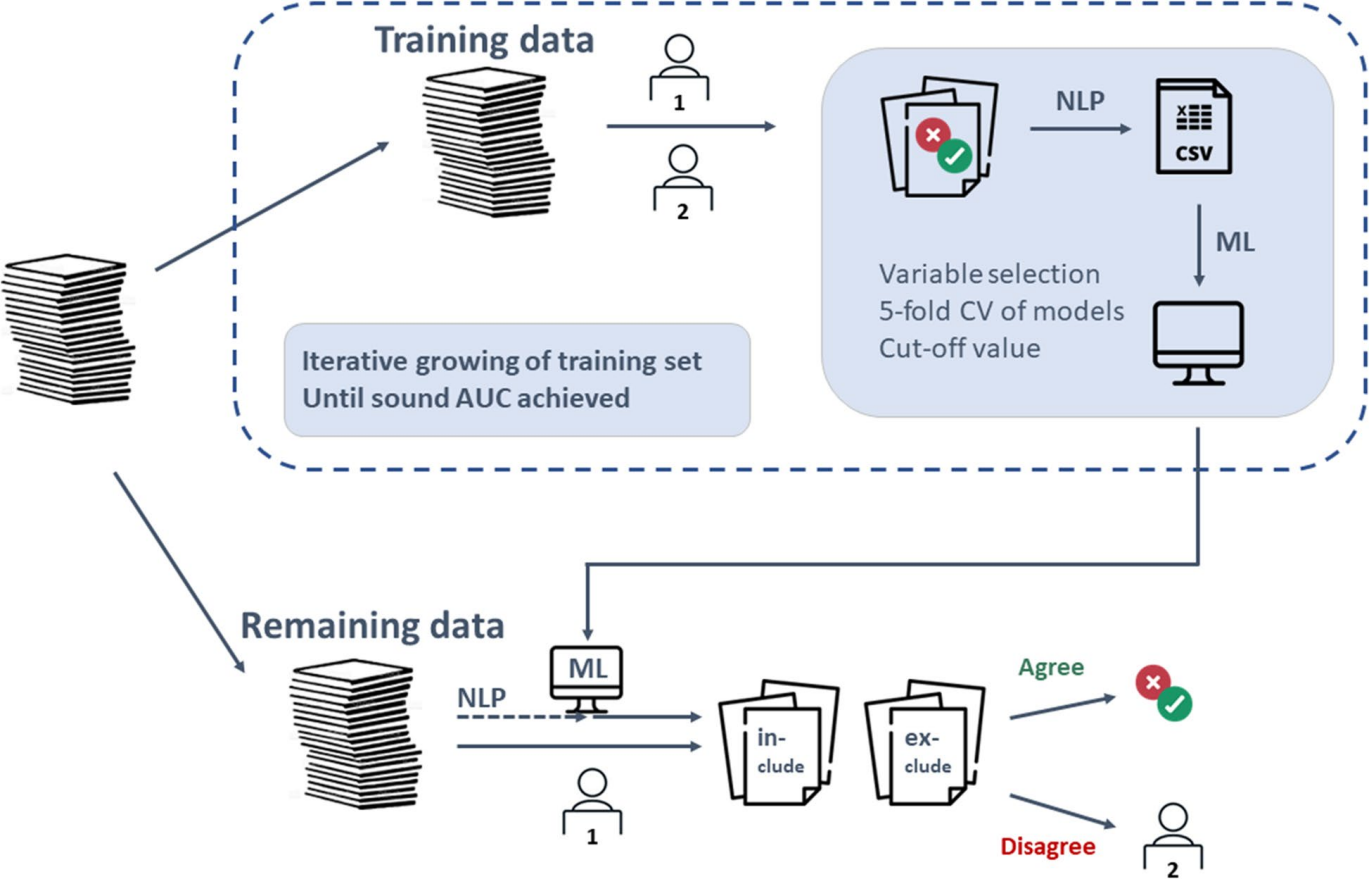
Extended workflow of presented methodology

Figure 1 showed the workflow of the presented ML approach. In practice, however, the picture has to be extended to demonstrate the entire workflow. This is depicted in Fig. 5. When applying the demonstrated approach in practice, a training sample has to be defined that is screened by two human reviewers. Based on this training sample, an ML model is trained as described above and a cutoff value is defined. Each publication in the test sample is forwarded to full-text screening if the model predicts an inclusion probability of at least this cutoff value. One human reviewer screens the citations in test sample as well. Only for decisions where the ML model and the human reviewer disagree, the second human reviewer is considered to solve the conflict.

### Size of training sample

The selection of the training sample size is critically important in practice. This consideration is particularly pertinent given the highly imbalanced nature of classes (inclusion/exclusion) in TIAB screening. Typically, there are significantly more manuscripts to exclude than to include. Consequently, it is impractical to recommend a single number as the optimal size of the training set. In some cases, even a large training sample may contain only a few publications to be included, which means that any ML model may lack sufficient information to accurately identify relevant publications. Thus, it may be more advisable to manually double-screen titles and abstracts until a certain threshold of to-be-included publications is reached. Setting this threshold is not trivial and may depend on the present classification task. For instance, one may think about increasing the training sample until the cross-validated AUC stabilizes. In our concrete example, we observe that the training sample should contain at least 69 inclusions (cf. the “Results” section). This number is obtained from Fig. 2 as the AUC stabilizes from a training sample size of 1341 on that corresponds to a number of included publications of 69. However, this value could be different in other examples such that a case-specific evaluation is recommended.

Moreover, an adaptive procedure, as employed by many existing software tools, may be worth considering. For example, human double-screening could be conducted until 70 included papers are identified. At this point, an ML model could be trained along with a cutoff value for inclusion. Subsequently, the ML model would be used to predict the inclusion probability for the remaining unscreened publications. Publications with inclusion probabilities near the cutoff value would be double-checked to further augment the training sample. The final ML model and cutoff value would then be determined based on this enlarged training sample.

### Illustration of cutoff value choice

To illustrate the workflow on a particular example, we choose the systematic review INTRISSI and assume that 40% of the data are available as training data. In this case, the training dataset consists of 1788 and the test set of 2672 publications. We trained a random forest on the training data and predicted the test set without knowledge of the outcome.

In the test set, there were 133 publications to be included and 2539 to be excluded for full-text screening, thus the data was quite imbalanced. Of note, this is not known in practice, as the test samples are not classified by two human reviewers. The cutoff value for inclusion to full-text screening is therefore selected based on the training sample exclusively. In the concrete example, selecting the cutoff value as the minimum of the cross-validated values described in the “Cutoff value” and “Choice of cutoff value” sections, yields a cutoff of 0.219. If in the test set, all publications with a to-be-included probability of at least 0.219 had been included, all publications that were included by human screeners were included by the ML approach as well. This would have implied that in total 1491 publications would have been included for full-text screening. Therefore, this cutoff allowed a true-positive rate of 1 and a true-negative rate of 0.465.

**Fig. 6:**
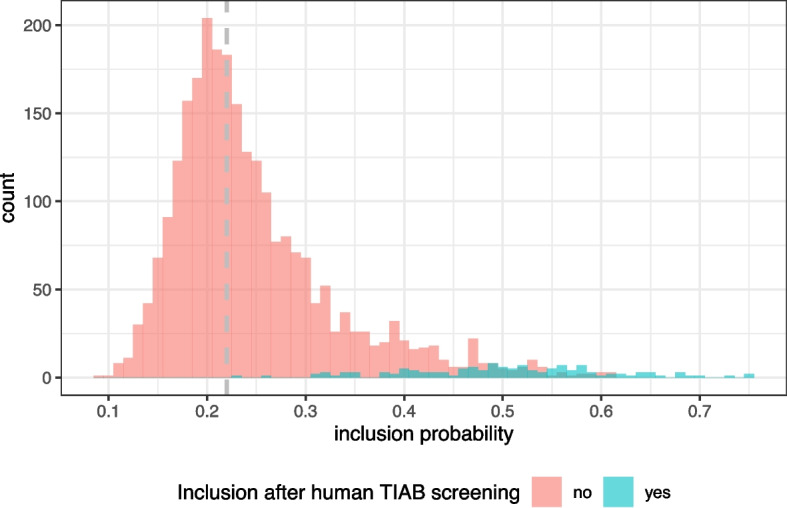
Histogram of inclusion probabilities of the test set

Figure 6 shows the inclusion probabilities for the test set. It demonstrates that by the cutoff value of 0.219 (gray line), all to-be-included publications would indeed be included. However, this comes with the price of including many false positive publications with a to-be-included probability close to the cutoff value. The larger the probability for inclusion, the less false positive cases appear. Choosing a larger cutoff of around 0.3 would save a lot of false positive results but imply two false negative results, i.e. two publications that should be forwarded to full-text screening would be excluded after title-abstract screening (by the decision of the ML model). Again, note that these values are not known in a real example as the test set decisions are not known.

The time savings by the ML approach can be analyzed by counting how many abstracts have to be read in the traditional and in the ML-based approach respectively. While in the traditional approach, two reviewers would read 4460 abstracts, in the ML approach, two reviewers would read 1788 abstracts until the ML model is sufficiently trained. The remaining 2672 abstracts would only need to be read by one reviewer. For the 1358 publications of the test set with contradictory decision between the ML algorithm and the human reviewer, a second human reviewer would be asked to read their abstracts. In total, 8920 abstracts would have been read in the traditional, and 7606 abstracts in the ML approach. Assuming that it takes 1 minute to read an abstract [44], this would result in saving 1314 minutes, i.e. more than 21 hours of reading time.

## Discussion

In this paper, we demonstrated that title-abstract screening can be partly automatized by an NLP and ML pipeline that converts the text into a dataset and treats the question of whether or not including a citation to full-text screening as a supervised classification problem. In one of the two given datasets, we showed that tree-based ML methods as well as fine-tuned language models can achieve AUC values of more than 0.95 when the training set consists of at least 1000 citations. This implies that human screeners only have to classify about 1000 publications while the remaining titles and abstracts could be allocated automatically to inclusion in or exclusion from full-text screening. It is not necessarily true that this generalizes to other title-abstract screenings as well and, therefore, we recommend a method comparison on the training set in order to identify the best ML method for the concrete problem at hand. In particular, different NLP or ML techniques than the presented ones may be more appropriate in another TIAB screening. The presented approach can be customized in a straightforward manner. Additional ML techniques can be implemented equivalently as in the supplemental software scripts.

A similar approach to this problem was presented by Kebede et al. [21], however without describing the NLP procedure in detail regarding lemmatization or stemming, which can have marked impact on the classification performance [17], and without the comparison to fine-tuned language models. They used more methods than we did in our approach and observed AUC values between 0.7 and 0.89. Their performance might be slightly worse since they did not include a general variable selection at the beginning and thus had to work with a larger amount of words. Similar to our work, tree-based approaches, in their case random forest and XGBoost, were among the best performing methods.

Fine-tuned language models showed a comparable to superior performance to classic ML algorithms, whereby it must be noted that no hyperparameter tuning was performed for runtime reasons and that we did not use the most recent language models. However, in the context of TIAB screening, language models suffer from some limitations. First, it takes a long time to fine-tune them what decelerates the training step and complicates tasks like cutoff computation. Second, since they use the titles and abstracts as a whole, they do not provide a variable importance measure. Therefore, the most important words for classification cannot be analyzed afterwards to validate the procedure. Seen through the lense of the emerging field of interpretable machine learning, the lack of such explanations may be seen as a limitation of language models. However, it is important to mention that the field of language models is growing rapidly and that the current front runners (for instance GPT-4) were not available for our analysis. When these models will be available for free in the future, they may outperform the methods presented in this manuscript. The same assertion holds for the possibility of hyperparameter tuning of language models, which may become better applicable in the future, if computer power increases. Of note, recent activities [45] present interesting ideas of how to overcome the limitation of runtime. There, the language models are not fine-tuned but the titles and abstracts of the training data together with the titles and abstracts to be classified are given within the prompt. This kind of prompt engineering enormously reduces the computation time and did not show inferior results for different language models [45].

Another important aspect with regard to the practical applicability of the presented approach is the combination of the NLP/ML pipeline with human screeners. Obviously, two human screeners are necessary to clearly classify the training sample of at least one thousand publications that will be used to train the ML algorithms. For the test data, different approaches are imaginable. First, as outlined in the “Practical implications” section, one may replace one human screener by the machine, thus the machine would serve as second reviewer. Another human screener would then be consulted to check cases in which the human and the machine decision differ. Second, to save more time, one may even imagine to classify the test sample completely by the NLP/ML engine. Publications whose inclusion probability are close to the defined cutoff value could then be analyzed by a human screener to reduce the risk to wrongly exclude important publications. This second approach could help to avoid time that is spent with publications that do not at all fit to the scope of the intended review, in particular with regards to the fact that a human screener needs on average one minute per title/abstract [44]. In total, the utilization scope of the presented approach depends on the individual risk one is willing to take to mistakenly miss few relevant publications.

In practice, the cutoff value that indicates whether a specific publication should be forwarded to full-text screening is of high importance. We presented an approach of how this cutoff value can be defined on the training set and evaluated it on the test set. Of course, such a test set evaluation is not possible in practice as the results of the test set are not known. The results from the “Choice of cutoff value” section showed that under a high-performing ML algorithm (in our results random forest or LightGBM), using the minimum of the cross-validated cutoff values as final cutoff value implies that no to-be-included publication in the test set is missed, even for a small training sample. Of course, this comes with the cost of a large number of false positive publications in the test set, i.e., publications that are falsely recommended for full-text screening. This can be toned down by two strategies. First, one may more deeply analyze publications whose to-be-included probability is close to the cutoff value in order to ensure correct decisions for them. Second, one can probably avoid a lot of these cases by including a second human reviewer in cases where the ML model and the remaining human reviewer disagree. We think that this strategy would detect the majority of false positive inclusions and thus reduce the amount of papers for full text screening. It is crucial to acknowledge that, regardless of the methodology employed, there is no assurance that all relevant publications will be captured. The fact that the presented strategies do not provide an estimate of the procedure’s recall (i.e., the proportion of correctly included publications among all publications) is a limitation of the described procedure and may be an interesting area for future research.

O’Connor et al. [46] discussed the barriers to adaption of automation tools. They claimed more evaluation and evidence were needed to gain trust in the systematic review community for the implementation of automation technologies. In the years that have passed since then, the evidence base has grown and tools were retrospectively evaluated and applied [14–16, 47]. Additionally, our approach proved to reduce the screening burden at minimal risk of missing any relevant study. With this in mind, we want to encourage the systematic review community, especially researchers with programming background, to use semi-automated title-abstract screening approaches in order to contribute to faster generation of systematic reviews with less workload. Even though there are several tools and platforms available, they are still very rarely used for systematic reviews [48].

For researchers it would be highly beneficial to not only automatize the title-abstract screening but also the full-text screening. However, this requires the application of the NLP techniques on a much larger amount of text, namely the entire paper and not only title and abstract. This implies more pre-processing work in order to correctly handle figures, tables, and further elements that appear between text blocks. Furthermore, this much larger number of words drastically increases the noise in the dataset and, therefore, makes the classification problem more difficult to solve. This is in particular important since one does not want to miss any publication that would have been included by human screeners. Despite these difficulties, the automatization of full-text screening is an interesting topic for future research when computer performance will improve.

## Conclusions

We presented the workflow to use natural language processing, machine learning, and fine-tuned language models to the task of title-abstract screening. The used methodology was described in detail and with focus on being reproducible in practice. The latter makes the approach easily adjustable to the particular needs of a specific systematic review and therefore facilitates its application. By means of a systematic review, we demonstrated the strong performance of these techniques by achieving AUC values of more than 95%. In a second systematic review, we could achieve AUC values of around 85% under smaller sample sizes. In both cases, it was illustrated that tree-based machine learning methods show a comparable performance to freely available fine-tuned language models. In particular, we extensively discussed a suitable choice of the cutoff value for inclusion of papers into full text screening in order to not only give a proof of concept but also give guidance how to apply the method in practice for future systematic reviews.

## Supplementary Information

Supplementary Material 1.

## Data Availability

The datasets used during the current study are available from the corresponding author on reasonable request. The R- and Python-code is available as supplemental material.

## Appendix: Tables

**Table 1:** AUC values with 95% confidence interval from different ML methods by size of training sample for the systematic review INTRISSI

Training size	Logistic regression	Support vector machine	Random forest	LightGBM	SciBERT	GPT-2
447	83 [80, 85]	78 [75, 82]	87 [85, 90]	88 [85, 90]	74 [70, 78]	55 [51, 59]
894	81 [78, 83]	79 [76, 82]	89 [87, 92]	88 [86, 91]	87 [84, 89]	78 [75, 81]
1341	79 [76, 83]	85 [82, 88]	95 [93, 96]	94 [92, 96]	93 [91, 95]	91 [89, 94]
1788	79 [74, 84]	89 [85, 92]	96 [95, 97]	95 [94, 96]	96 [95, 97]	92 [90, 94]
2235	83 [78, 87]	92 [90, 95]	96 [95, 97]	95 [94, 96]	97 [96, 98]	96 [95, 97]
2680	80 [75, 85]	91 [88, 94]	96 [95, 98]	95 [93, 97]	97 [96, 98]	94 [92, 96]
3125	81 [75, 87]	77 [71, 84]	97 [96, 98]	93 [90, 96]	97 [96, 98]	96 [95, 98]
3570	80 [72, 87]	90 [85, 94]	97 [96, 99]	93 [89, 97]	98 [97, 99]	96 [95, 98]
4015	78 [66, 90]	83 [72, 94]	97 [95, 99]	96 [92, 99]	97 [95, 98]	96 [93, 98]

Values are given as a percentage

**Table 2:** AUC values with 95% confidence interval from different ML methods by size of training sample for the systematic review PPU

Training size	Logistic regression	Support vector machine	Random forest	LightGBM	BERT	GPT-2
135	75 [70, 81]	63 [56, 71]	71 [63, 78]	67 [60, 73]	70 [64, 76]	52 [46, 59]
270	72 [67, 78]	60 [51, 68]	69 [62, 76]	73 [67, 80]	79 [74, 84]	54 [48, 60]
405	72 [66, 78]	57 [48, 66]	68 [58, 77]	68 [58, 78]	77 [71, 83]	64 [57, 70]
540	68 [59, 76]	61 [51, 71]	75 [67, 84]	74 [65, 83]	76 [69, 83]	73 [66, 80]
674	69 [61, 76]	60 [50, 70]	77 [68, 86]	80 [72, 87]	91 [88, 94]	76 [69, 83]
808	59 [47, 71]	59 [47, 71]	81 [73, 89]	76 [67, 84]	87 [82, 92]	80 [73, 88]
942	65 [52, 78]	62 [49, 75]	78 [65, 91]	81 [71, 90]	88 [83, 94]	84 [76, 92]
1076	72 [59, 86]	68 [56, 80]	83 [70, 96]	80 [68, 93]	92 [87, 97]	84 [75, 94]
1210	70 [43, 97]	66 [43, 89]	76 [52, 100]	80 [56, 100]	89 [75, 100]	85 [65, 100]

Values are given as a percentage

## Abbreviations

ML: Machine learning
NLP: Natural language processing
SVM: Support vector machine
TIAB: Title-abstract
